# Occult intraocular aluminium foreign body causing rhegmatogenous retinal detachment: a case report

**DOI:** 10.1186/s12886-023-02881-w

**Published:** 2023-03-30

**Authors:** Ling Zhang, Bin Chen, WeiMin He

**Affiliations:** 1 Ophthalmology Department, West China Hospital, Sichuan University, No. 37 Guoxue Lane, Wuhou District, Chengdu City, Sichuan Province China; 2 Ophthalmology Department, The People’s Hospital of Leshan, No. 238 Baita Street, Shizhong District, Central District, Leshan City, Sichuan Province China

**Keywords:** Aluminium intraocular foreign body, Rhegmatogenous retinal detachment, Occult, Ocular trauma

## Abstract

**Background:**

Ocular trauma is complex and varied, and some occult intraocular foreign bodies (IOFBs) can lead to uncommon symptoms and signs. We report a case of rhegmatogenous retinal detachment (no obvious wound, no pain, no intraocular infection or other symptoms) caused by an occult intraocular aluminium foreign body, which could have been easily missed.

**Case presentation:**

A 42-year-old male presented to the outpatient department of our hospital complaining of fluttering black dots and decreased vision in his left eye that began 3 months earlier. He was diagnosed with "floaters" at a community hospital. He denied a history of ocular trauma or previous surgery. The cornea and lens of the left eye were clear. A small patch of pigmentation was noted in the temporal sclera. Fundoscopy revealed macula-off retinal detachment. After mydriasis, elliptical holes were seen in the peripheral retina at 2:30, and a suspicious hyperreflective strip was found under the anterior lip of the retina by Goldmann three-mirror contact lens examination; the strip was confirmed to be an IOFB by orbital CT. The IOFB was removed through pars plana vitrectomy without any complications.

**Conclusion:**

Unlike iron and copper IOFBs, aluminium IOFBs are more inert and more likely to be missed. For people with special occupations (construction workers, mechanics, etc.), when abnormal pigmentation of the sclera is found, the possibility of foreign bodies in the eye should be considered. In the process of disease diagnosis and treatment, it is necessary to ask for a detailed history, including occupation history and practice, and perform careful physical and targeted examinations. Such comprehensive analysis regarding the above information will minimize the chance of missed diagnosis.Awareness of occult IOFB in high risk occupations and prompt referral to a retinal surgeon is of outmost importance.

## Introduction

Intraocular foreign bodies (IOFBs) are common ocular injuries in the working population. In addition to direct injuries caused by foreign bodies penetrating the eyeball, different secondary injuries, such as infectious endophthalmitis, uveitis, and traumatic cataract, can also occur. Iron and copper ions can cause damage to the crystalline lens and retina, and nonmetallic foreign bodies can cause chronic inflammation [1]. We report a case of an occult intraocular aluminium foreign body causing rhegmatogenous retinal detachment without the typical signs of the presence of an IOFB.

## Case report

**Fig. 1:**
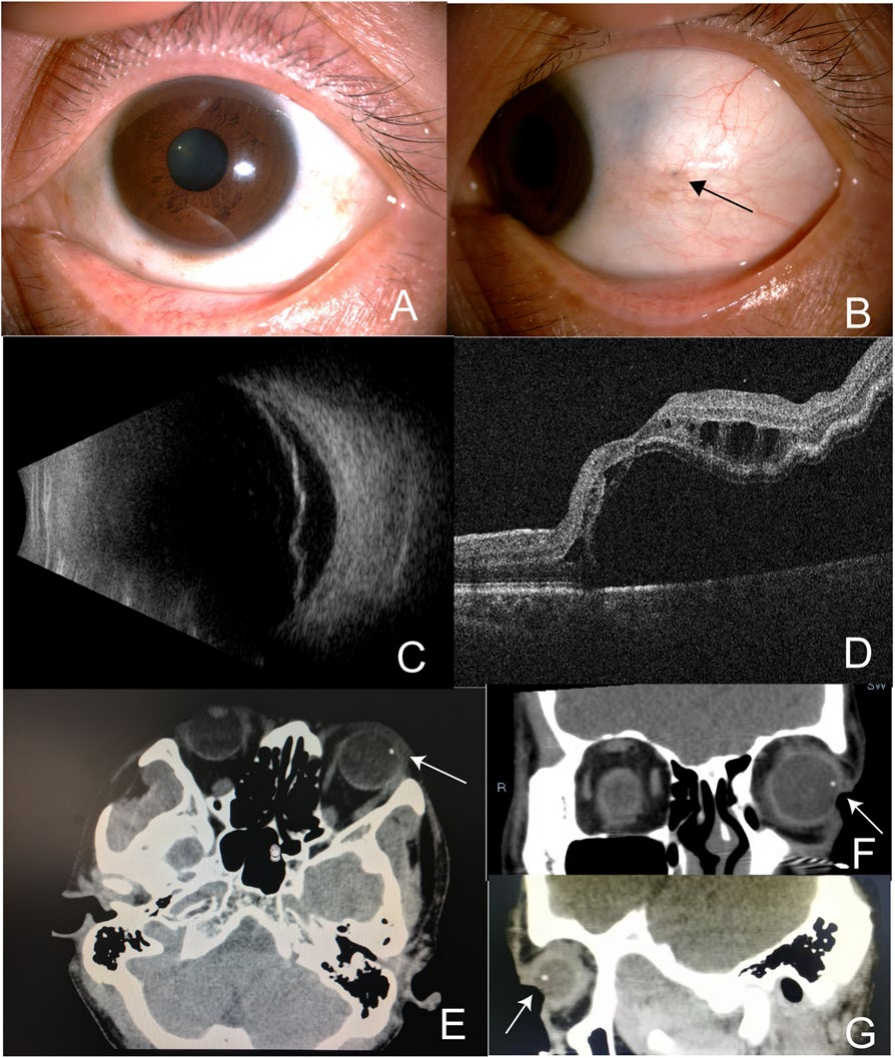
**A** shows that the corneal lens is clear without an obvious penetrating wound; The arrow in **B** shows a small patch of pigmentation in the temporal sclera, which was presumed to be the point of entry of a foreign body into the eyeball; **C** B-ultrasound showing retinal detachment; **D** OCT showing retinal detachment; the vitreous cavity was clean without pigmentation or bleeding; **E** horizontal axis-CT showing an intraocular high density foreign body shadow (indicated by the arrow); **F** coronal axis-CT scan showing an intraocular high density foreign body shadow; **G** sagittal axis-CT showing an intraocular high density foreign body shadow

**Fig. 2:**
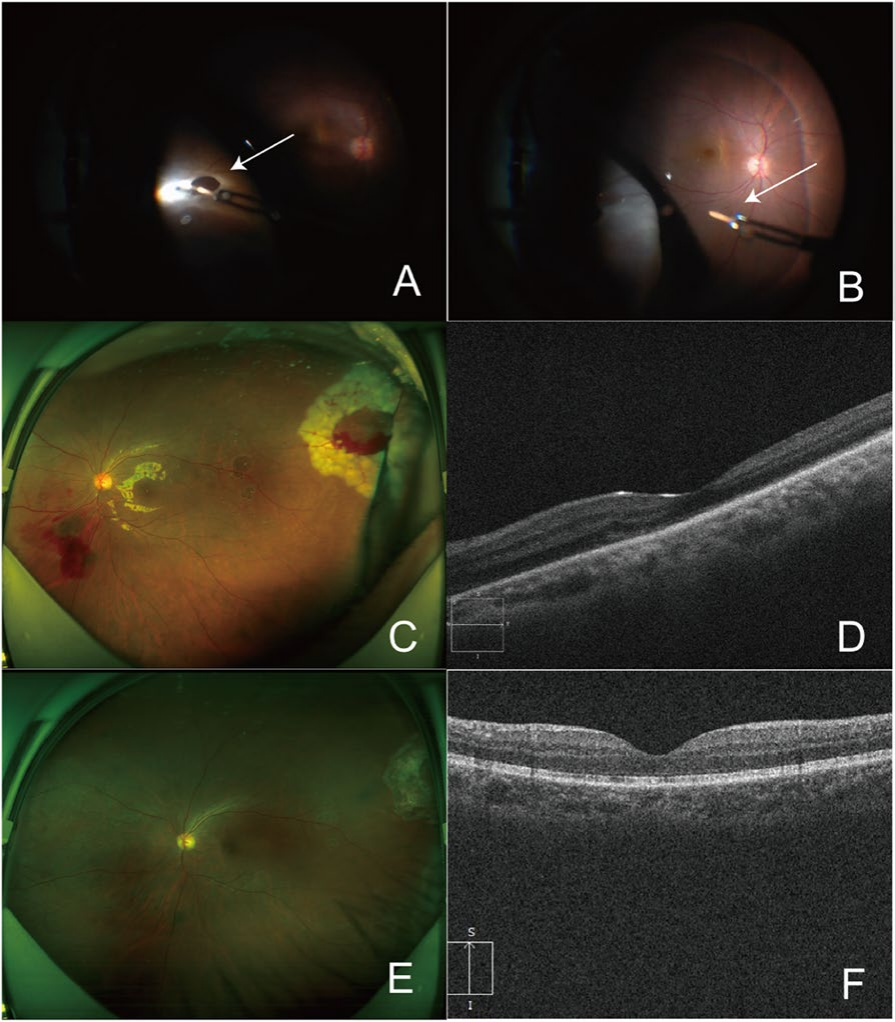
**A** and **B** show the intraocular foreign body removed from the retinal tear during vitreous surgery (the arrow in A indicates that the intraocular foreign body was located external to the retina); **C** and **D** show retinal reattachment on the third postoperative day; **E** and **F** show that the retinal and macular structures had recovered well 1 year after the operation

The patient, a 42-year-old male, came to the hospital in December 2019 because of "fluttering black dots and decreased vision in his left eye for 3 months". He was diagnosed as having "floaters" at a community hospital. His symptoms worsened despite 2 months of oral medication (lecithin-bound iodine < Jolethin > , 1.5 mg orally 3 times/day), and he was referred to our hospital. His general condition was normal, and his past medical history was unremarkable. He denied a history of ocular trauma or previous surgery. The physical examination results were as follows: The best-corrected visual acuity was 20/20 in the right eye and 1/20 in the left eye. The intraocular pressure was 18 and 14 mmHg in the OD and OS, respectively. The anterior and posterior segments of the right eye were normal. The cornea and lens of the left eye were clear, and there was no sign of a penetrating injury wound. A small patch of pigmentation was noted in the temporal sclera (Fig. 1A, B).Vitreous hemorrhage +  + and pigment + . Temporal retinal detachment between 12 and 4 o’clock involving the macula was observed. B-ultrasound and OCT examinations of the eye indicated retinal detachment only (Fig. 1C, D). A dilated fundus examination with a Goldmann three-mirror contact lens revealed an elliptical peripheral retinal hole (a single hole is seen in the image) with a suspicious subretinal hyperreflective strip at 2:30 o’clock, which was confirmed to be an intraocular foreign body by orbital CT (Fig. 1E–G). Upon further questioning, even though the patient denied a definite history of ocular trauma, he admitted to engaging in cutting and grinding aluminium metal objects in his long-term vocation as a mechanic. We therefore speculated that the patient may have been injured by the high-speed sputtering of fine broken metal pieces caused by the cutting machine. Because the foreign body was small and entered the globe at a high speed, it did not cause significant eye discomfort. Blood tests, liver function tests, kidney function tests, and other preoperative tests were within normal limits. In January 2020, the patient underwent vitrectomy of the left eye combined with removal of the intraocular foreign body under local anaesthesia. During the operation, a fine strip of a white metallic foreign body was successfully removed under the tear hole of the anterior lip of the retina (Fig. 2A, B). It was a nonmagnetic metal (due to his long history of cutting and grinding aluminium metal objects, we concluded that it was aluminium), approximately 0.5 × 3 mm in size. After surgery, the patient was treated with tobramycin, dexamethasone and levofloxacin topical eye drops. He was followed regularly in the outpatient department, and his latest examination at one year after surgery was as follows: visual acuity was 12/20 in the left eye, and retinal reattachment was good (Fig. 2C-F).

## Discussion

Intraocular foreign body injury is a common type of open globe ocular trauma. Due to its special pathogenic mechanism, it is listed separately in the classification of mechanical ocular trauma [2]. It is easy to diagnose based on patient history of ocular trauma and characteristic ocular manifestations. However, its diagnosis can be easily missed in cases with an unknown history of ocular trauma and atypical ocular manifestations [3]. Common clinical manifestations are mainly due to secondary complications, such as uveitis [1], endophthalmitis [4], secondary glaucoma [5], ocular siderosis [6], and traumatic cataract [7]. Multiple signs can be present at the same time. Some studies defined patients with occult intraocular foreign bodies as those with an unclear history of ocular foreign body injury or a history of ocular foreign body injury accompanied by ocular damage but negative imaging examination results [8]. Even if there is no definite ocular wound and the patient does not remember any history of trauma, the clinical presentation and occupational characteristics of the patient can help to diagnose intraocular foreign bodies [9, 10]. In this patient, anterior segment examination of the left eye was normal apart from a small patch of pigmentation, which was noted in the temporal sclera. We speculated that the foreign body penetrated the eye from the temporal sclera and caused the retinal tear, while the cornea and lens remained uninjured. Because the foreign body was a small and narrow, nonmagnetic material (presumably aluminium or an aluminium alloy) and possibly entered the eye at a high temperature and high speed after processing by the cutting machine, it did not cause intraocular infection or inflammatory reaction. Unlike metals such as iron and copper, aluminium is an inert metal, so it does not cause obvious damage to eye tissues such as the retina and crystalline lens.

This case underlines the importance of obtaining a detailed history, including occupation history and practice, and performing careful physical and targeted examinations, including dilated funduscopy in all patients complaining of floaters. At the same time, awareness of the occurrence of occult IOFBs in individuals working in high-risk industries needs to be raised. Such comprehensive analysis of the above information will minimize the likelihood of misdiagnosis. Any patient who is suspected of having an IOFB should be referred to a retinal surgeon. If the foreign body in the eye is an inert metal,it may present with atypical clinical signs. Vitreous surgery to remove the foreign body in a timely fashion improves the visual prognosis.

## Data Availability

All the data supporting our finding is contained within the manuscript.
